# Improved drug therapy: triangulating phenomics with genomics and metabolomics

**DOI:** 10.1186/s40246-014-0016-9

**Published:** 2014-09-01

**Authors:** Andrew A Monte, Chad Brocker, Daniel W Nebert, Frank J Gonzalez, David C Thompson, Vasilis Vasiliou

**Affiliations:** 1 University of Colorado Department of Emergency Medicine, Leprino Building, 7th Floor Campus Box B-215, 12401 E. 17th Avenue, Aurora 80045, CO, USA; 2 Skaggs School of Pharmacy and Pharmaceutical Sciences, Aurora 80045, CO, USA; 3 Rocky Mountain Poison & Drug Center, Denver 80204, CO, USA; 4 Laboratory of Metabolism, Center for Cancer Research, National Institute of Cancer, Bethesda 20892, MD, USA; 5 Division of Human Genetics, Department of Pediatrics and Molecular Developmental Biology, University of Cincinnati Medical Center, Cincinnati 45220, OH, USA; 6 Department of Environmental Health and Center for Environmental Genetics, University of Cincinnati Medical Center, Cincinnati 45220, OH, USA

**Keywords:** Individualized medicine, Genomics, Metabolomics, Omics, Personalized medicine, Phenomics, Systems biology, Transcriptomics

## Abstract

Embracing the complexity of biological systems has a greater likelihood to improve prediction of clinical drug response. Here we discuss limitations of a singular focus on genomics, epigenomics, proteomics, transcriptomics, metabolomics, or phenomics—highlighting the strengths and weaknesses of each individual technique. In contrast, ‘systems biology’ is proposed to allow clinicians and scientists to extract benefits from each technique, while limiting associated weaknesses by supplementing with other techniques when appropriate. Perfect predictive modeling is not possible, whereas modeling of intertwined phenomic responses using genomic stratification with metabolomic modifications may greatly improve predictive values for drug therapy. We thus propose a novel-integrated approach to personalized medicine that begins with phenomic data, is stratified by genomics, and ultimately refined by metabolomic pathway data. Whereas perfect prediction of efficacy and safety of drug therapy is not possible, improvements can be achieved by embracing the complexity of the biological system. Starting with phenomics, the combination of linking metabolomics to identify common biologic pathways and then stratifying by genomic architecture, might increase predictive values. This systems biology approach has the potential, in specific subsets of patients, to avoid drug therapy that will be either ineffective or unsafe.

## Introduction

Recent advances in genomics, epigenomics, transcriptomics, proteomics, metabolomics, and phenomics have allowed identification of certain factors associated with variable drug responses. However, with few exceptions, high-fidelity prediction of drug efficacy and safety on a larger scale has proven elusive. We have experienced many failures with pharmacogenetic attempts to predict success of drug therapy; this is due to insufficient knowledge and oversimplification of experimental approaches, as well as failure to accept that even the simplest traits almost always reflect an intersection of multiple genetic, epigenetic, and environmental factors. Therefore, we make an argument for embracing complexity. Starting with a clinically relevant trait—the individual patient drug response, which we refer to as ‘phenotype’—and then working backward to integrate biological data, is more likely to identify common pathways and inter-individual variability between patient responses. We propose that this integrated-systems biology approach, focused on drug response and adverse drug reactions (ADRs), might be the best way to improve drug efficacy and safety.

## Limitations of single biological associations

‘Personalized medicine’ has endured many failures. Clinical phenotype of complex diseases (defined as any biological, physiological, morphological, or behavioral trait) is difficult to predict because most common diseases represent multifactorial traits.

Use of pharmacogenomics in predicting drug efficacy or toxicity is more advanced in oncology than perhaps in any other area of medicine. A high degree of efficacy and toxicity can be predicted for chemotherapeutic agents in cancer, based partially upon patient and tumor genotyping. For example, 5-fluorouracil toxicity is associated with dihydropyrimidine dehydrogenase polymorphisms [1],[2], erlotinib [3] or cetuximab [4] responsiveness is linked to epidermal growth factor receptor polymorphisms, and there are other examples [5]. However, because tumor cells mutate or the patient develops associated co-morbidities (e.g*.* renal failure, pulmonary hypertension), then efficacy and toxicity remain difficult to predict. Focusing on a single biological association can sometimes efficiently predict drug response within a subset of a large cohort of patients, but rarely, if ever, can we expect to predict drug response in the individual patient [6].

## Genotype-phenotype associations

Virtually, all clinical traits are polygenic, raising the number of potential phenotypes factorially; even the simplest genetic predictors lead to a range of phenotypes. This was demonstrated in 1960 when the earliest pharmacogenomic association of polyneuropathy with slow acetylation of isoniazid by *N*-acetyltransferase-2 (NAT2) was reported [7]. Not all individuals with the slow-acetylator trait develop neuropathy, and there remains a range of serum drug concentrations within each group of *NAT2* genotypes. Currently, at least 190 different *NAT2* alleles have been identified, and these polymorphisms result in a large range from “no effect”, to slow, to very efficient acetylation [8]. In addition, *NAT2* performs AcCoA-dependent *O*-acetylation of *N*-hydroxyarylamines and AcCoA-independent *N*,*O*-acetylation of *N*-hydroxy-*N*-acetylarylamines, which suggests a pleiotropic effect of this polymorphism, likely contributing to variable penetrance of the phenotype [9]. Prediction of the expressed phenotype is more complex than initially appreciated, due to the wide range of downstream modifications and environmental factors that can function to alter expression of the underlying genetic code—leading to the ultimate biological response.

If prediction of success is defined as ‘high positive and high negative predictive value of the test’, then all single biological associations have failed miserably. Strong biological associations can be made in small homogenous cohorts; in larger, more diverse populations, however, these associations are inevitably poor predictors of phenotype.

The literature is replete with examples of small cohort or observational studies, later shown in larger prospective trials to be poorly predictive; *CYP2C9* and vitamin K oxidoreductase complex subunit-1 (*VKORC1*) polymorphism-guided warfarin anticoagulation is foremost among such examples. Several early studies demonstrated benefit to *CYPC9* and *VKORC1* genotyping for prediction of ‘time in the therapeutic window’ and ‘dose’ of warfarin [9]–[12]. These correlations did not hold up in larger randomized controlled trials [13]–[15].

## Genome-wide association studies

Genome-wide association (GWA) studies have demonstrated a strong association between HLA-B*1502 and carbamazepine-associated Stevens-Johnson Syndrome and toxic epidermal necrolysis in Asian [16], but not in European populations [17]. The epigenomic drug vorinostat, a histone deacetylatase inhibitor, was initially found to profoundly decrease lymphoid proliferation in human cell lines [18], but demonstrated only a 30% response rate in a small cohort, prior to US FDA approval for cutaneous T-cell lymphoma [19]. Initial studies had suggested that transcriptomic screening for rejection following cardiac transplantation might be more sensitive than endomyocardial biopsy [20]. In a larger prospective trial, fewer biopsies were necessary in transcriptome-tested patients, although this did not eliminate the need for biopsy and only 6 of 34 rejection episodes were identified by the gene profiling test [21].

Testing for such single biological associations is therefore clinically impractical, due to such poor predictive values. Single biological associations should not be viewed as predictive; failure should be expected because of the extraordinarily large number of steps required to yield a phenotype after drug ingestion [5],[6],[22],[23]. Specifically, the amount of drug absorbed may vary due to intestinal metabolism or disease, unpredictable hepatic blood flow can change the rate that enzymes metabolize the drug, polymorphisms in transporters and metabolic enzymes can lead to variable amounts of drug delivered to the systemic circulation, the drug must be transported to the site-of-action, and the site-of-action itself may be altered by polymorphisms en route to the observed clinical response, and the rate of elimination may be variable due to altered renal clearance [5]. This is further complicated in multifactorial traits, which increase potential phenotypic variability.

## Starting with phenomics

Phenomics, defined as the unbiased study of a large number of expressed traits across a population, is the logical starting point for biological association studies. Traditional biological experiments, such as GWA studies, begin with the investigator selecting a phenotype and then attempting to associate biological differences within a population. Significant resources have been devoted to characterize the individual patient’s whole genome, epigenome, transcriptome, proteome, metabolome, as well as gut microbiome. This approach has been robust; GWA studies have identified more than 4,000 polymorphisms linked to more than 500 clinical traits [24]. Unfortunately, it remains possible that identified single-nucleotide variants (SNVs) are not the causative factor but rather are associated with some additional metabolic factor that is polygenic. This effect-modification phenomenon has been recognized for decades in epidemiologic studies [25].

Ultimately, the unequivocal phenotype is what patients and clinicians wish to predict. Therefore, studies should shift focus away from these single biological associations that are only statistically associated with a phenotype when studying a large cohort, to an approach that characterizes the biologic system starting with phenotypic variables. Initiating analysis from a wide number of characterized traits [termed phenome-wide association (PheWA) studies] allows the investigator to see the intertwined biological processes leading all the way back to genetic associations.

Phenomics captures pleiotropic associations (genes associated with several traits) that are often overlooked [26]. Examples include *HLA8.1* association with both myasthenia gravis and thymus hyperplasia [27] (and likely other autoimmune diseases), as well as lipid-gene associations with plasma glucose and insulin resistance [28]. Whereas two traits may both be associated with the same SNV, this association does not provide unequivocal information about the relationship between the traits.

Some traits may be the direct result of SNVs while others are secondarily associated, thereby confounding the relationship. The aldehyde dehydrogenase-2 (ALDH2) Glu504Lys polymorphism is a classic example of this phenomenon; this amino-acid change is associated with a disulfiram-like reaction in Asians [29], one of the strongest genotype-phenotype associations. This polymorphism can also be associated with alcohol intolerance, but this is not the causal phenotype of the *ALDH2* polymorphism. Instead, a decrease in ALDH2-dependent metabolism is the associated phenotype, and decreased tolerance is likely due to the unpleasant disulfiram-like reaction rather than an inability to develop alcohol tolerance [30]. The clinically-relevant phenotype of alcohol intolerance may be modified by co-linearity with other metabolism enzyme gene polymorphisms such as *CYP2E1*[31],[32], with genotypic factors dictating body mass index [33], and/or with addictive behaviors [34].

Examination of a wide range of phenotypes can identify which pathways are causal, and which are secondary effects. Specialized analysis techniques for PheWA studies must be utilized because phenomic data structure varies significantly, comprising binary as well as continuous variables when compared with other large datasets such as GWA studies [35],[36]. At present, we are not aware of any phenomics studies that have been applied to drug therapy, although we predict this will happen soon. Detailed electronic medical record (EMR) data will allow for large-scale phenomic studies, when paired with GWA studies or other biological databases [37].

Phenomics demands a systems biology approach. Phenotypes must be associated with biological pathways, which in turn reflect genomic architecture. For example, a systems biology approach, starting with the trait ‘sepsis survival’, has identified new metabolic pathways characterizing survival [38]. This breakthrough gives us a more comprehensive understanding of the metabolite markers of survival as well as numerous genetic contributors, thereby permitting identification of biomarkers and potential therapeutic targets. Lack of a full understanding of all involved biological pathways can lead to false prioritization of phenotypes. For example, for years, the ‘cataplexy’ phenotype in rodents was targeted for the development of neuropsychiatric drugs. Unfortunately, this narrow focus appears to have led to the development of more drugs that produce extrapyramidal symptoms than provide antipsychotic efficacy [39].

## Adding metabolomics to your arsenal

Metabolomics is the study of small molecules in biologic fluids or tissues. This methodology has been used to discover metabolites that act as diagnostic tools for a growing number of medical conditions. For example, metabolite profiles from human tissue, urine and plasma accurately predicted differentiated benign prostate hypertrophy from clinically-localized prostate cancer and metastatic disease [40]. In this case, complex and overlapping clinical phenotypes are tightly correlated with specific changes in metabolite homeostasis, which could then be further segregated into precise, distinguishable disease states. A similar scenario has been seen for metabolomic studies of ovarian cancer progression [41]. Moreover, urinary and serum biomarkers may also serve as a non-invasive and rapid means of identifying and monitoring phenotype-metabotype relationships during the onset and progression of disease [42],[43]. Performed in an unbiased manner, addition of metabolomics to systems biology has provided insight into gene networks, resultant metabolic changes, and genotype-phenotype relationships [44]. These studies highlight the need for unbiased metabolomics to link phenomic data with genomic data.

## Metabolomics: linking genomics with phenomics

Small molecules identified by metabolomics represent the substrates, intermediates, and products of all biochemical pathways. Consequently, this technique represents an integrated snapshot-in-time of all upstream biologic processes, ultimately leading to a clinical phenotype. Therefore, the metabolome may be the closest biological representation of a clinical trait. Indeed, metabolomics has identified possible biomarkers for cardiac [45], kidney [46], liver [47], and gastrointestinal [48] diseases, as well as many other pathophysiological conditions.

Metabolomics profiling is incredibly sensitive and can detect femtomolar to attomolar changes in metabolite concentrations [49]. Anything from small dietary changes, increased physical activity, elevated stress, or even seasonal variables can significantly alter the composition of metabolomic profiles. This profound sensitivity has obvious advantages: small metabolomic changes can account for complex physiological changes. However, because of increased sensitivity, specificity may be sacrificed, thus decreasing the predictive value of the test.

In some cases, large metabolic changes are able to maintain stability of a clinical trait; these findings can identify potential areas of metabolic decompensation. For instance, elevated lactate is a marker of impaired gluconeogenesis, as are increased concentrations of other gluconeogenic substrates, such as pyruvate and alanine, which are preferentially metabolized to lactate [50],[51]. Mildly elevated levels of these metabolites are not clinically significant, but instead represent compensated buffer activity. If further stress is placed on the system, lactic acid itself can overwhelm the buffered system and lead to decompensated metabolic acidosis with high mortality [52]. Metabolic changes, such as lactic acidosis, can be the result of numerous upstream responses; post-exposure monitoring may predict severe disease processes and ADRs prior to phenotypic manifestation.

Therefore, the metabolome may be viewed as an overly sensitive tool with insight into adaptive physiology [53]. This sensitivity can serve to identify a wider range of genomic polymorphisms that contribute to expressed phenotypes, even if only under specific circumstances. Unbiased metabolomics has identified novel pathways in hypertension that include microbiome associations and sex steroid mediators previously not appreciated to contribute to the hypertensive phenotype [54]. Metabolomics identified the mechanism of anti-retroviral drug interaction mediated by the CYP3A4 enzyme through newly recognized enzymatic functions [55]. These mechanisms have implications for choosing drug therapy in HIV patients. The presence of five amino acids was correlated with the development of diabetes as well as an insulin-resistant phenotype in a cohort of initially normoglycemic patients followed for 12 years [56]. Similar findings identified new pathways in insulin resistance identified by the oral glucose tolerance test [57]. Metabolomics was able to predict poor response to insulin; thus, metabolomics has potential to predicting development of disease and therapeutic success [58]. Metabolomics can be a powerful tool to elucidate complex mechanisms of drug-modified clinical traits [59].

## Genomic architecture

Despite the claim that pharmacogenetics has ‘at best, a marginal benefit’ [60], genetics represents a constant in any biological system on which we can build models and stratify expected phenotypes. As noted above, limitations of GWA studies must be recognized, but stratification of clinical traits can be accomplished with a systems biology approach. Pharmacogenetics may allow stratification of starting doses for drugs and identification of patients who are unlikely to respond at the recommended prescribed dose or who are at risk for serious ADRs.

Elucidation of such ‘extreme discordant phenotypes’ (EDPs) [61] is useful to physicians because EDP methodology can lead to decreased healthcare expense and lower patient morbidity. Seventy-four percent of all physician office visits involve drug therapy. In 2008, prescriptions accounted for $234.1 billion in patient costs [62]. Forty-eight percent of people in the USA take at least one prescription; more than 76% of people 60 years and older take two or more [62]–[64]. Despite the remarkable number of prescribed drugs, many of these medications are ineffective. For instance, hypertension is the most common chronic disease in the USA, yet 64% of patients receiving antihypertensive therapy fail to achieve blood pressure control [65]. Another factor beyond the scope of this review is the issue of ‘non-compliance’: the patient failing to adhere to his prescribed drug regimen. Non-compliance can easily be misinterpreted by the investigator as drug failure, thereby leading to increased ‘noise’ in any study of genotype-phenotype associations [6]. This confounder can be addressed by metabolomic screening to confirm drug ingestion [66].

If patients can be stratified with an EDP approach and clinical phenotypes modeled using sensitive metabolomics, it may be possible to reduce the number of office visits for ineffective therapy or ADRs. However, virtually, all clinical traits are multifactorial, with each haplotype contributing only a small fraction (0.1%–0.0001%) to the ultimate phenotype. For example, in a GWA study of almost 184,000 subjects, 180 loci associated with ‘height’ as the trait were identified; however, these loci together contributed only approximately 10% to variation in the phenotype [67]. This underscores the need to account for downstream modifying factors, and to assess the individual as a sum of his phenotypic parts.

Another major problem in all genotype-phenotype association studies is that our present-day methods can capture only additive genetic variance, which probably accounts for 70%–80% of heritability for any multifactorial trait. The remaining 20%–30% represents non-additive genetic variance. At this time, we have no methodology for finding this ‘missing heritability’ [*cf.*[68],[69] for further details].

## Triangulating phenomics with genomics and metabolomics

Combining metabolomic and GWA databases has resulted in identification of new biological pathways in cardiovascular, kidney disorders, type-2 diabetes, cancer, gout, venous thromboembolism, and Crohn’s disease [70]. Phenomics has not yet been overlaid upon these analyses. Thus, important pleiotropic clinical traits with common biological pathways remain to be discovered.

By viewing each phenotype as a node within a systems biology model, linked by metabolic pathways and dictated by genetic architecture, we can more fully understand the relationship between biological processes that result in variable clinical traits. Proof of this concept has been demonstrated in the KORA F4 cohort that identified several SNVs associated with type-2 diabetes by way of metabolomics association studies [71]. Preliminary metabolomic studies have also proved useful in helping to differentiate phenotypically heterogeneous disorders, such as Parkinson’s disease (PD). PD can present as either slowly or rapidly progressive forms, which can be distinguished early by comparing metabolite profiles. Identified metabolites might subsequently be used to shed light on the underlying metabolic abnormalities and pathway perturbations driving phenotypic variation [72]. Only by embracing the complexity of the biological system can we improve predictive accuracy.

## Combining transcriptomics and metabolomics

Other methodologies will undoubtedly need to be integrated. For instance, transcriptome analysis using RNA sequencing (RNA-seq) can be used to quantitatively identify global changes in gene expression in tissues and serum. Integration of transcriptomics and metabolomics data allows accurate identification of genes and enzymatic pathways driving downstream alterations in metabolite distribution, production, and degradation. These data can provide profound insight into a given phenotype by positioning biochemical compounds within metabolic pathways as substrates, intermediates or products. In addition, compensatory mechanisms can be identified during pathway analysis and help explain the absence of an expected phenotype. Integration of transcriptomics-metabolomics data provides a means to track how metabolic pathways change, in response to disease and/or therapy, and then directly tie those alterations with phenotypic outcomes.

**Figure 1:**
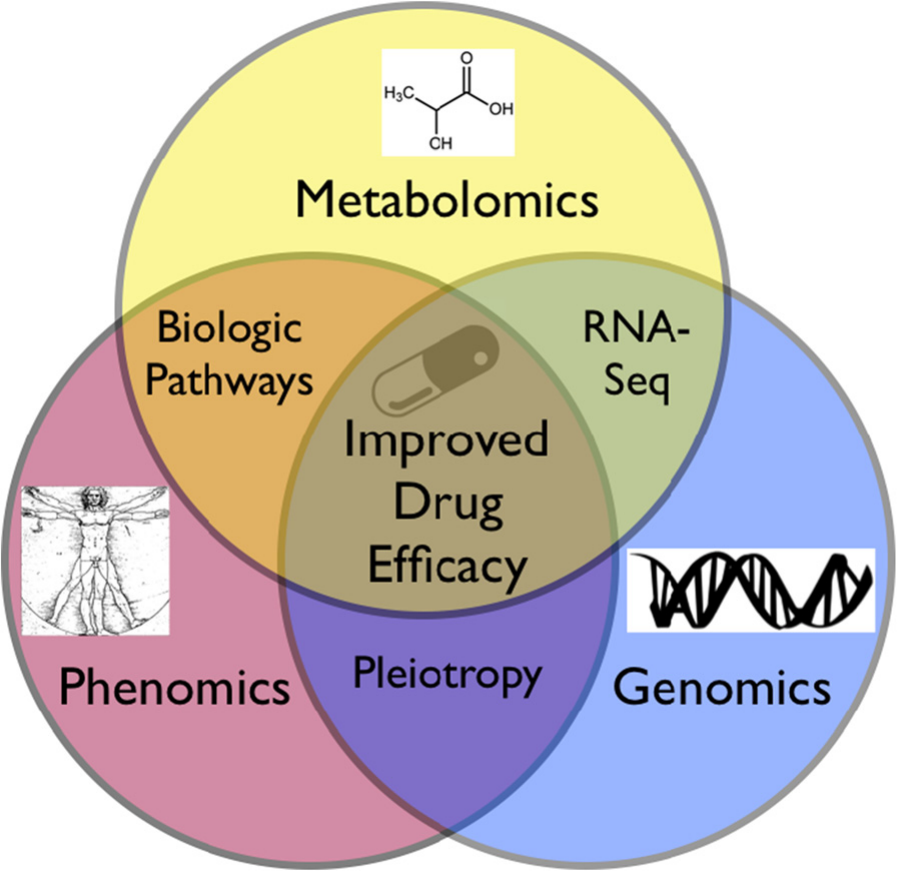
**Illustration of an integrated systems biology approach to improve drug therapy.** Using phenomics to fully characterize clinical traits associated with drug therapy. When combined with metabolomics, common biological pathways can be identified, providing insight into mechanisms of efficacy and safety. When phenomic data associated with genomics data are also combined, pleiotropic associations can be further identified and contribute to our understanding of underlying biological pathways. Other techniques, such as RNA-seq, can be integrated to add depth to the pathway data and supplement our understanding of genomic expression.

Multilayer systems biology is still an emerging field, but initial studies using combined transcriptomics and metabolomics data look promising with regard to identifying phenotype-genotype-metabotype relationships (Figure 1), including those observed during pancreatic cancer progression, melanoma response to chemotherapy, as well as others [73]–[77].

Technically, the biggest bottleneck confronting the utilization of extremely large combined datasets is the difficulty in analysis and processing bioinformatics. In other words, there can be drawbacks to adding too much data. We will likely experience an ‘hourglass effect’ as we integrate more variables into predictive models. Initially, more data may improve such a model. As too many factors become integrated into the model, overfitting becomes problematic and the model becomes inherently unstable, sacrificing predictive accuracy. This phenomenon has been well documented in climate modeling. This argues for simplification of the model variables at points distant from the phenotypic outcome, as well as elimination of factors that contribute only marginally. New software systems, such as GenAMAP have been developed specifically for this purpose. GenAMAP allows for integration of genomics, transcriptomics, and phenomics data accounting for differences in dataset structure when one uses multivariate structured association-mapping algorithms [78].

## A clinical example

**Figure 2:**
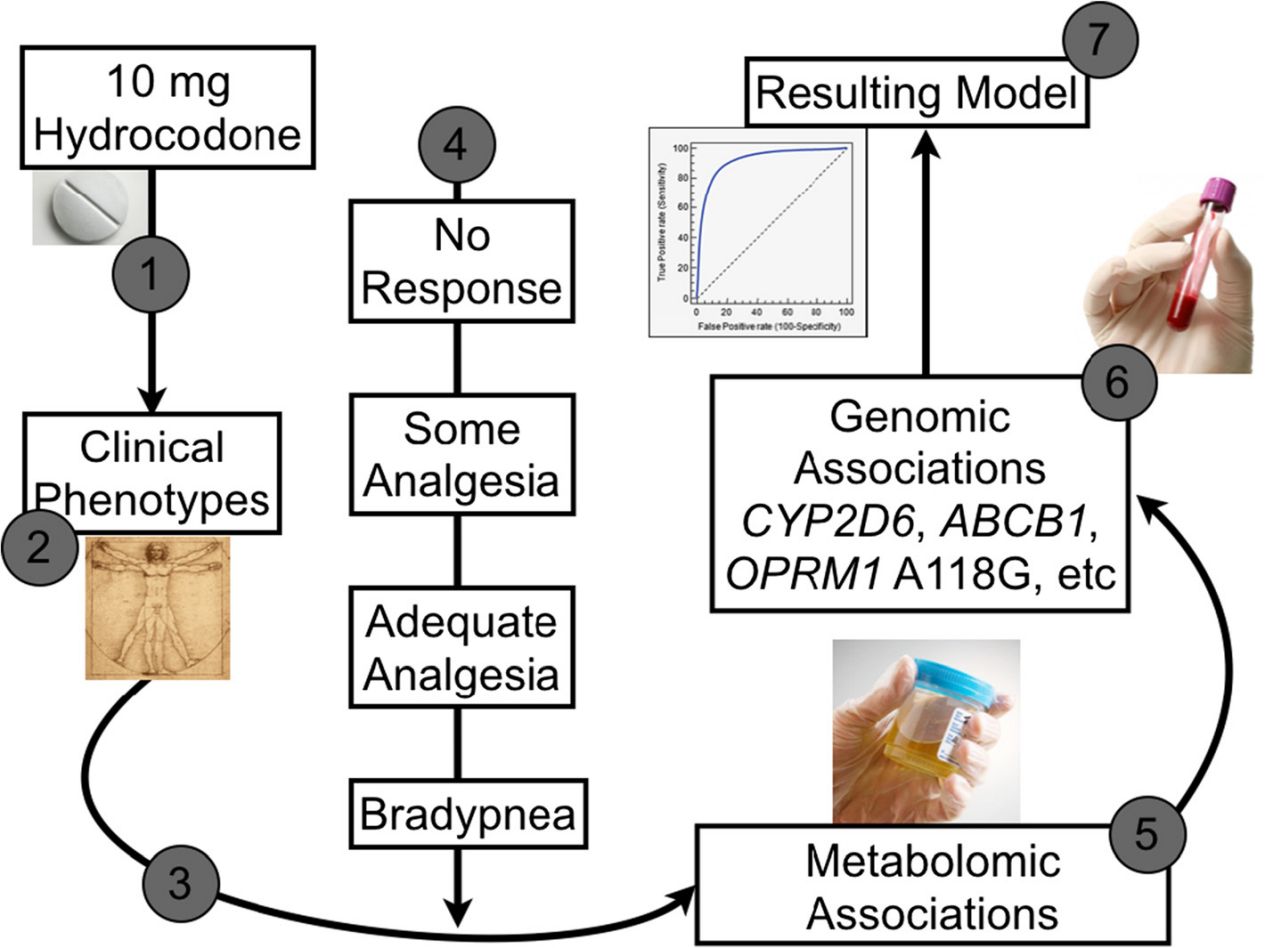
**A clinical example of the proposed integrated method for predicting drug response.** (1) Patients are given 10 mg of hydrocodone. (2) Clinical phenotypes are captured fully and completely. These may include (among others) development of ADRs, chronicity of treatment, ethnic differences, and demographic factors. (3) Association studies may contribute to characterization of the clinical phenotypes (e.g. RNA-sequencing may help distinguish chronicity of treatment). (4) The drug response is categorized into phenotypically pertinent groups. (5) Relevant biological pathways are identified and linked by individual metabolomic markers. (6) Stratification of drug response is refined by accounting for biological pathway polymorphisms and controlled for phenotypic variables captured in #2 above. (7) The final stepwise model is built, allowing for a high, although not perfect, receiver operating characteristic (ROC).

Ideally, the approach starts with phenomics, then is stratified by genetic associations, and finally, further refined with metabolomic associations. This will require following thousands of patients with full characterization of biological samples and clinical outcomes for every commonly prescribed drug. For example, if we consider hydrocodone efficacy and safety phenotypes, we can build a model based on genetics/genomics and metabolomics factors. Consider patients given 10 mg of hydrocodone with careful characterization of phenotypic clinical responses and ADRs (Figure 2). Adding RNA-seq transcriptomic data from serum may allow determination of human opioid *mu*-1 receptor expression (*OPRM1*), which would differentiate chronic users from naïve users. Metabolomic analysis in pre- and post-drug-challenged patients may identify factors associated with efficacy, ADR phenotypes, and genetic architecture known to be associated with hydrocodone variability in response. Clinical responses can be stratified by genetic factors such as *CYP2D6* polymorphisms, principle metabolic pathways for hydrocodone activation associated with active drug levels [79], the multi-drug resistance transporter-1 (*MDR1*; official name *ABCB1*) that transports many opioid drugs [80], and *OPRM1*, in which 118A/A homozygous patients exhibit a correlation with hydrocodone pain relief [81]. Again, metabolomics associations may provide markers of these polymorphisms and identify the pertinent pathways involved, as well as new previously unrecognized pathways that might serve to identify additional associations.

Ultimately, this stratification of data can be distilled into an integrated model using programs that raise the receiver operator characteristic (ROC) curve for determining rate of efficacy and risk of ADRs. Some clinical factors may contribute to the ultimate drug-response phenotype, to varying degrees between patients. For instance, stomach acidity may affect drug dissolution; this complex trait (among other factors) can be influenced by inter-individual gene expression, concomitant drug therapy, and even the time of the day. Consequently, it should be obvious that specific factors may contribute significantly to the ultimate phenotype in some patients, provide only a minor contribution in others, and only contribute under certain circumstances (e.g*.* suppression of stomach acid with a proton pump inhibitor), in a third group. Because many of these complex relationships are present in each drug-patient interaction, a perfect predictive model will never be possible.

## Conclusions

Whereas perfect prediction of efficacy and safety of drug therapy is not possible, improvements can be achieved by embracing the complexity of the biological system. Starting with phenomics, linking metabolomics to identify common biologic pathways, and stratifying by genomic architecture—this combination can increase predictive values. We believe that this systems biology approach has the potential to eliminate drug therapy that will either be ineffective or unsafe, in specific subsets of patients.

## Competing interests

The authors declare that they have no competing interests.

## Authors’ contributions

All authors contributed knowledge and expertise in the writing of this review. All authors read and approved the final manuscript.
